# Dementia Caregivers’ Emotional Language in Relationship Narratives and Associations with Well-Being

**DOI:** 10.1093/geroni/igaf122.4235

**Published:** 2025-12-31

**Authors:** Jenna Wells, Dustin Gad, Emily Mroz

**Affiliations:** Cornell University, Ithaca, New York, United States; Cornell University, Ithaca, New York, United States; Emory University, Atlanta, Georgia, United States

## Abstract

Dementia drastically alters caregivers’ relationships with their care recipients, and these changes can erode caregivers’ well-being. Although relationship narratives offer a rich window into individuals’ subjective impressions of their relationships, a narrative sharing approach to data collection is rarely applied in the context of dementia caregiving. The present study asked 67 current caregivers of people with dementia to verbally narrate two defining memories from their relationship with their care recipient from: (1) during caregiving and (2) before caregiving began, and to complete self-report questionnaires of their current well-being. We used Linguistic Inquiry and Word Count (LIWC-22) to quantify caregivers’ use of positive, negative, anger, sadness, and anxiety language in their relationship-defining narratives. Pearson correlations indicated that greater anger language in the “before caregiving” narratives was associated with higher depressive symptoms; greater sadness language in the “during caregiving” narratives was associated with higher anxiety symptoms and higher premorbid (pre-caregiving) relationship closeness; and greater positive language in the “before caregiving” narratives was associated with higher premorbid relationship closeness (rs = .25-.30, ps < .05). No associations were found with anxiety language in either narrative type. Caregivers who express anger about their pre-caregiving relationship may feel conflicted about their current caregiving role, contributing to increased depressive symptoms. Additionally, caregivers who felt more connected to the care recipient prior to dementia may express more sadness as their relationship changes, which in turn may fuel anxiety symptoms. Future research should evaluate emotional language as a potential target for narrative-based interventions to improve dementia caregivers’ well-being.

